# Enhancing quantitative capacity for the health sector in post-Ebola Liberia, a tracer study of a locally developed and owned coding and biostatistics program

**DOI:** 10.12688/f1000research.154839.1

**Published:** 2024-09-02

**Authors:** Snoyonoh T. Barcon, Trokon O. Yeabah, Mulbah K.A. Kromah, George B. Davis, Laura A. Skrip

**Affiliations:** 1 Quantitative-Data for Decision-Making (Q4D) Lab, Monrovia, Liberia; 2 Division of Infectious Disease and Epidemiology, National Public Health Institute of Liberia, Monrovia, Liberia; 3 Liberia Institute of Statistics and Geo-Information Services, Executive Mansion Ground, Monrovia, Liberia; 4 School of Public Health, College of Health Sciences, University of Liberia, Monrovia, Montserrado, Liberia

**Keywords:** quantitative analysis, capacity strengthening, health system strengthening, Liberia, meaningful outcomes, evidence-driven decision-making

## Abstract

**Background:**

Despite the demonstrated value of quantitative research in understanding and responding to public health events, analytics capability is not always prioritized or available in settings that would greatly benefit from it. In Liberia, there are no university degree-granting programs in biostatistics or mathematical modeling, promoting dependence on external technical assistance. To address the gap, a local NGO, Quantitative-Data for Decision-Making (Q4D), was founded to enhance capacity and opportunities for analyzing quantitative data among Liberians.

**Methods:**

To understand the relevance, utility, and impact of the skills being taught at Q4D, a tracer study was undertaken with current and former students. Participants completed an online survey that evaluated how often and in what ways they are applying course skills, as well as any personal or professional advancement they have attributed to their learning of coding and/or biostatistics through the program.

**Results:**

Among 43 participants, 81% reported a high level of confidence in independently applying skills learned through Q4D classes in their jobs and/or academic programs. Most participants (81%) responded that they were actively demonstrating the skills they acquired; 74% were teaching the skills to others. Among the 83% of employed participants who reported using the skills currently in their jobs, 56% rated the skills they learned as very or extremely useful in their current positions. Several students attributed salary increments, consultancy opportunities, and scholarships to the skills gained through the program.

**Conclusions:**

Program skills are being applied by students employed in health-related sectors, suggesting that the training content is relevant and useful for addressing some of the workforce’s analysis needs. Moreover, skills built through the program have positively impacted students by preparing them with the skills required for additional employment and training opportunities to advance in-country health research capacity and reduce inequities.

## Background

Situated in West Africa, Liberia has experienced back-to-back shocks due to decades of civil conflict, followed by the unprecedented Ebola outbreak ^1^ and ongoing public health threats, including COVID-19, mpox, measles, and Lassa fever. ^2^^–^^4^ Despite efforts to enhance resilience across sectors, including with the adoption of a pro-poor agenda, ^5^ the country presently ranks 177 (out of 191) on the Human Development Index. ^6^ Largely external investments in capacity-building have sought to fill gaps. ^7^ For the health sector, institutional developments have improved systems to better prepare the country for detecting, notifying, and responding to threats in the post-Ebola period; however, investments in improving data quality, analysis, and use have been less prioritized than the generation or reporting of data, and systems remain vulnerable. ^8^

Despite Liberia’s experiences with the 2014-2015 Ebola outbreak and more recent COVID-19 pandemic and the extensive quantitative research undertaken with external collaborators to understand the potential trajectories of these health crises, ^9^^–^^11^ few efforts have been made in Liberia to institutionalize local capacity strengthening opportunities in quantitative data analysis for public health researchers and practitioners. To date, there is no university degree being administered in biostatistics, and dynamic mathematical modeling is not taught even as a course in existing academic programs. Short-term training often facilitated by visiting collaborators is being conducted at institutional levels. However, the limited literature being produced in Liberia using quantitative analysis ^12^ and the reliance on international consultants for work that is being done ^13^ provide evidence that the few existing models of teaching coding and statistics may not be generating independent capacity. That gap has implications for overall development as well as resilience in the face of health and other threats. ^14^^–^^16^

To provide a local opportunity for health sector personnel to undertake advanced and sustained analytical learning, Quantitative-Data for Decision-Making (Q4D) Lab was developed by a team of local and international public health scientists. The NGO envisions training Liberians who are working in, or aim to work in, the health sector in relevant quantitative skills. The existing course series introduces foundational coding skills in R Statistical Software ( https://www.r-project.org/), followed by bivariable analysis and visualization, and ultimately multivariable regression with generalized linear models. An alternative route provides training in dynamic transmission modeling after foundational coding and statistics skills are developed. Most students engage with Q4D after learning about the program from peers who have attended classes. The student body reflects people working at the Ministry of Health, the National Public Health Institute of Liberia, international non-governmental organizations, as well as students in undergraduate and graduate public health programs or in health professions schools. The classes have nominal fees ( *e.g.*, $50USD for Beginner R class which is less than the fee of one credit hour at the local public university), making them accessible but also attaching some value to hold students accountable to attending sessions. Moreover, class sessions focus on practice versus theory to keep the content and skills grounded in actual applications in the Liberian health sector context—both the health issues facing the sector and the constraints facing those attempting to conduct research everyday. ^15^^,^^17^

**Figure 1.**
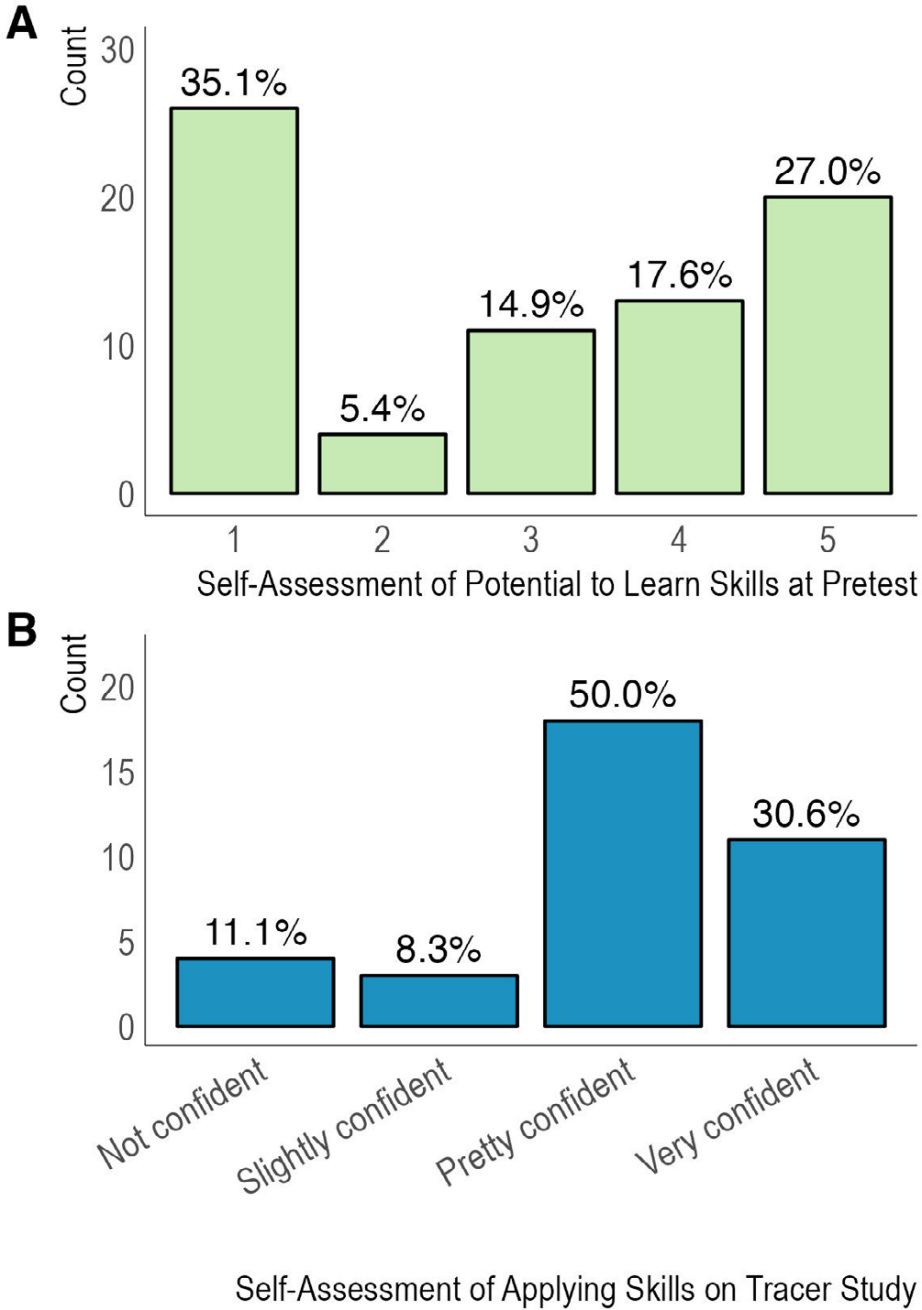
Confidence expressed by students around their abilities to learn and apply coding skills in R. Legend. Panel A reflects programmatic data (not collected as part of this study) from a Beginner R course pretest on how students rated their confidence in being able to develop coding skills in R, with 1 being not at all confident and 5 being extremely confident. Panel B displays data from the Tracer Study on how students rated their confidence in independently practicing the R coding and/or Biostatistics skills they learned after participating in Q4D courses.

Based on an understanding of the limited foundation for coding and statistics generated by current educational programs in Liberia, ^17^ the Q4D Beginner R class assumes no working knowledge of R or Biostatistics and builds confidence and skills from the ground up. Programmatic data offers insight into students’ baseline, with students reporting ahead of the Beginner R program that they were not too confident that they could develop coding skills in R software (35% rated themselves 1 or ‘not at all confident’ out of 5 on a confidence scale, with a median ranking of 3) ( Figure 1A) and that they anticipated their lack of previous analysis and coding experience would be a challenge to successfully completing the program. Thus, despite often being already employed in the health sector and having roles related to monitoring and evaluation, most students enter the Q4D program with limited computer skills and little or no prior experience with coding or statistical software (When asked if they had previously encountered a problem that needed to be solved using a spreadsheet or data analysis, nine out of ten indicated that they had and that they had been unable to solve it.). While the Q4D program was developed with this contextual understanding, no study to date has been undertaken to evaluate whether the skills taught in the program are fulfilling the goals of Q4D Lab in terms of their relevance to data and analytics needs in the health sector and their utilization outside of the classroom. Specifically, to inform programmatic growth and larger scale evaluations, it is important to gauge whether the capacity being built is being used by students in their personal and professional lives and is perceived as having impact.

This paper thus aims to highlight contributions that the Q4D program has made towards quantitative analysis capacity in Liberia, specifically in the health sector. The findings offer insight into the utility and relevance of the course content for current workforce needs and reflect how the skills gained have contributed to personal or professional advancement of students to date.

## Methods

### Study design and rationale

A cross-sectional tracer study approach was used to collect data from students who had engaged with Q4D course content. The approach was largely descriptive and intended to understand if and how students were using skills taught in the course(s), and the perceived relevance and utility of such skills to the health sector and to students’ personal and professional growth. Outcomes of interest were thus experienced outside of the classroom, in contrast to skills assessments where performance is the outcome or process evaluations where acceptability and feasibility of program design are outcomes. Future iterations of the study would allow for more longitudinal follow up.

### Study setting and participants

The Q4D office and classroom are based in Monrovia, the capital city of Liberia. Monrovia is mostly urbanized and is recognized as a center of social, economic, and political activities, with the population of Monrovia (approximately 1.74 million) representing one-third of the total population in Liberia. Most Q4D students are working in public institutions or with non-governmental organizations based in Monrovia. Many local universities, including the nation’s largest public university, are also in or near Monrovia.

At the time of data collection (June-July 2023), Q4D had enrolled 54 unique students, each attending one or more of the classes offered. Three of the students had been hired for Research Associate positions with the program and were excluded due to their professional and financial engagement with the Q4D program. The remaining 51 students were eligible to participate based on the inclusion criteria of enrollment in at least one of the Q4D courses and attendance of at least three class sessions, as some enrolled students did not complete courses due to scheduling conflicts or other factors. Q4D courses range in duration from 10 total class sessions (for Beginner class) to 16 total class sessions (for Advanced classes).

### Data source

A researcher-developed questionnaire was programmed into KoboCollect for electronic data collection (See Extended data). In addition to sociodemographic background questions about participants’ education level and employment status, the questionnaire included largely closed-ended questions that evaluated how often and in what ways participants are applying the skills learned during the Q4D courses, including whether they are telling, showing, and teaching the skills they learned to others. Specific examples of skills applications were solicited via open-ended questions. The questionnaire was also intended to gauge the impact that the coding and analysis skills are having on current and future professional and academic experiences of students. Particularly, information was collected on whether the skills gained contributed to career growth or other personal and professional development. Lastly, participants were asked about the impact that the capacity being built by the program could have on the health sector.

### Data collection process

An invitation email with an attached consent form and a link to the online questionnaire was sent to each of the 51 eligible students; potential participants also received a randomly assigned 3-digit code that they were asked to enter upon indicating consent to participate. The code was used for tracking purposes to maintain confidentiality (no names or contact information were requested in the questionnaire) and for preventing follow-up communication being sent to those who already completed the questionnaire. After a week, follow-up attempts were made via email, WhatsApp and direct phone call to encourage potential participants to check their email for information about the study and consider completing the questionnaire.

### Sources of bias

The tracer study was an evaluation of the Q4D approach to capacity strengthening. To avoid potential bias in presentation of findings due to self-evaluation, data collection and data analysis procedures were conducted in collaboration with authors TOY and MKAK who were not previously involved with Q4D but who are data and statistics experts in Liberia. Additionally, eligibility criteria were set to include participants who may have dropped from classes before certification to capture perspectives of individuals who may have found the skills too challenging or inconsistent with their expectations when enrolling in the class. This was aimed to reduce bias of only including participants who successfully completed the courses and may have had more positive experiences.

### Variables and data analysis

Responses to survey questions were summarized descriptively using frequencies and percentages. Variables of interest included demographic characteristics and employment (health sector or other), how quantitative capacity developed through Q4D programs was being told, shown, and/or taught to others, the relevance and utility of skills taught through the program, and personal and professional growth opportunities that participants attributed to their engagement with the Q4D program. All descriptive statistics and data visualization were done using R Statistical Software version 4.3.0.

### Ethics statement

The study protocol was approved by the University of Liberia IRB (ULIRB IORG-IRB Number: IRB00013730) on May 16, 2023. All study procedures were carried out according to the Declaration of Helsinki. Participants were asked to provide written consent via an online form ahead of gaining access to the questionnaire. The consent form text is included in the Extended data. All results have been presented using aggregate statistics rather than any individual participant’s response. For direct quotes on specific examples of skills applications, potentially identifying information—such as on health district where one works—was removed.

## Results

### Overall study sample

A total of 43 unique participants responded to the tracer study survey, representing a response rate of 84.3% (43/51). Respondents reflected students who had attended one or more of the four courses currently offered, including the Beginner R coding class (n=40), the Biostatistics I with Intermediate R course (n=19), the Biostatistics II with Advanced R course (n=8), and the Mathematical Modeling with Advanced R class (n=3). The Beginner R class is a prerequisite for other classes unless students can demonstrate they have foundational coding skills in R. Among participants, reported rates of course completion were 80.0% (32/40) for Beginner R, 89.5% (17/19) for Biostatistics I, 87.5% (7/8) for Biostatistics II, and 33.3% (1/3) for the Mathematical Modeling course.

Most participants were male (33/43, 76.7%) ( Table 1). At the time of the survey, 45% of participants held a Bachelor’s degree as their highest educational attainment (19/42), while 48% indicated having a Master’s degree (20/42). One respondent is a PhD holder and another has completed a postdoctoral fellowship. More than 40% of respondents indicated being currently enrolled in a degree program (18/42, 42.9%), primarily graduate programs at the Master’s or PhD level. Nearly three-fourths of participants indicated being employed either part-time or full-time.

**Table 1.** General characteristics of study sample.

Characteristics	Overall (n=43) *
**Sex**	
Male	33/43 (76.7)
Female	10/43 (23.3)
**Highest education level completed**	
Bachelor	19/42 (45.2)
Medical Doctor (MD)	1/42 (2.4)
Master	20/42 (47.6)
Doctor of Philosophy (PhD)	1/42 (2.4)
Postdoctoral fellowship	1/42 (2.4)
**Current enrollment in a degree program**	
Bachelors	2/42 (4.8)
Masters	13/42 (31.0)
PhD	3/42 (7.1)
**Current employment status**	
Part-time	3/41 (7.3)
Full-time	27/41 (65.9)
**Description of employer** **	
International NGO	8/29 (27.6)
Local NGO	1/29 (3.4)
Public/government	16/29 (55.3)
Private for-profit company	3/29 (10.3)
Other	1/29 (3.4)
**Sector of employment** **	
Health	20/30 (66.7)
Non-Health	10/30 (33.3)

* Data presented as n/N (%) unless otherwise indicated. For each characteristic, N excludes those who responded “Don’t know” or who chose not to answer, unless otherwise indicated.

** N reflects those who are currently employed and responded to the question.

Most employed participants reported working for public or governmental institutions (16/29, 55.2%), while others responded working for international non-governmental organizations (NGOs) (8/29, 27.6%), a local NGO (1/29, 3.4%), or for-profit, private sector institutions (3/29, 10.3%). Furthermore, two-thirds of those employed indicated working in the health sector (20/30, 66.7%).

### Changes in quantitative capacity available to the health sector

Participants reported high confidence in their abilities to independently practice the skills taught during the Q4D program at work or at school. About 81% responded being “pretty confident” or “very confident” in independently practicing the skills at their job and as part of a study program, while 8% were “slightly confident” in independently applying the data analysis skills learned ( Figure 1B). This reflects a shift in confidence among students in their ability to learn coding and analysis skills relative to their self-assessment at the pretest ( Figure 1A).

**Figure 2.**
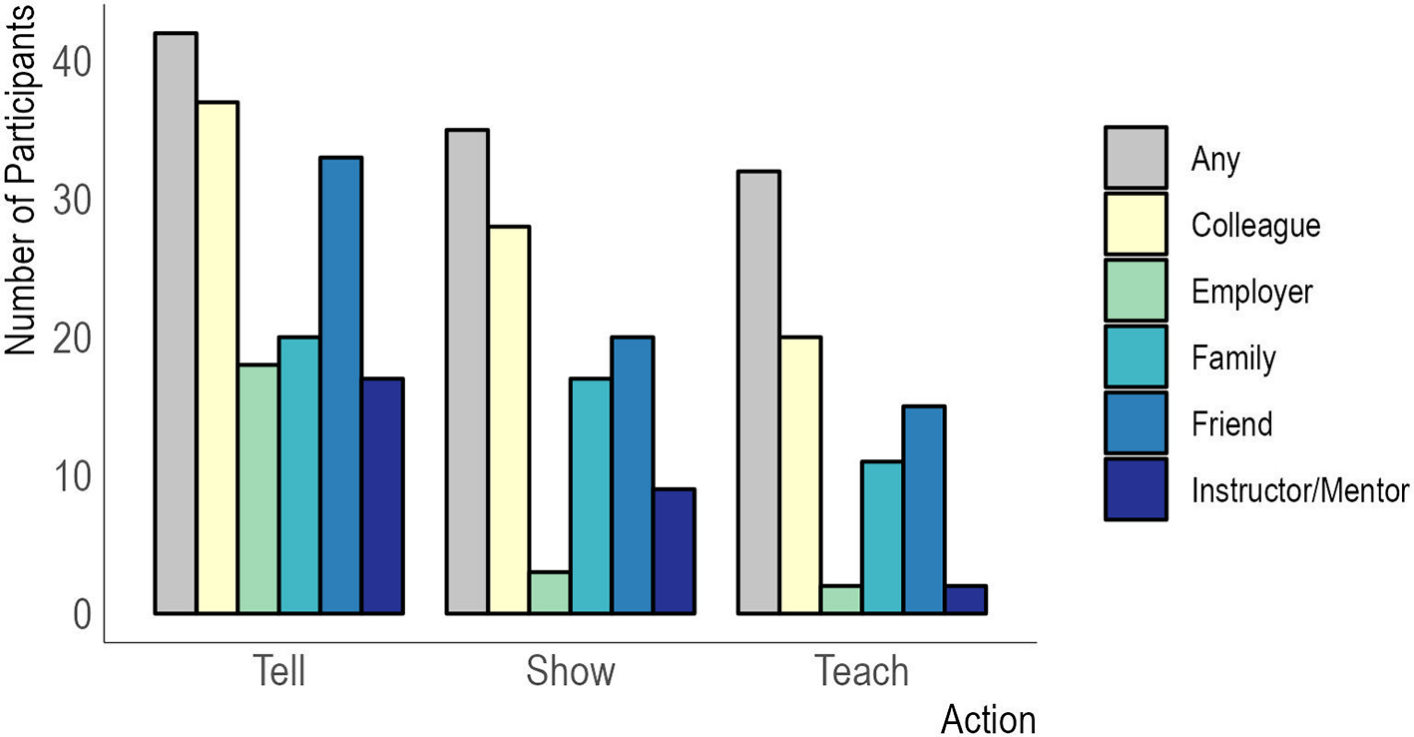
Persons told, shown, or taught R coding and analysis skills by Q4D students. Legend. Survey participants indicated whether they had shared the skills they were learning at Q4D with different people in their lives as metrics of their excitement and confidence in the course content, as well the cascading of skills to other people not directly in the program.

Students reported that they are telling others about their R skills, demonstrating the skills, and/or teaching others the skills. Almost all the participants (97.6% 42/43) reported that they have shared information about the skills with friends, colleagues, employers, family members, or mentors/instructors ( Figure 2). Furthermore, around 81% (35/43) have demonstrated their skills to others, and 74% (32/43) have taught the skills learned at Q4D to friends, colleagues, employers, family members, and/or mentors/instructors. When asked to rate the degree to which the skills taught at Q4D might impact decision-making within the health sector of Liberia, students indicated a median rating of 9 out of 10 (IQR: 8-10), with 10 representing ‘the highest’ level of impact.

### Relevance and utility of skills to current professional or academic activities

**Figure 3.**
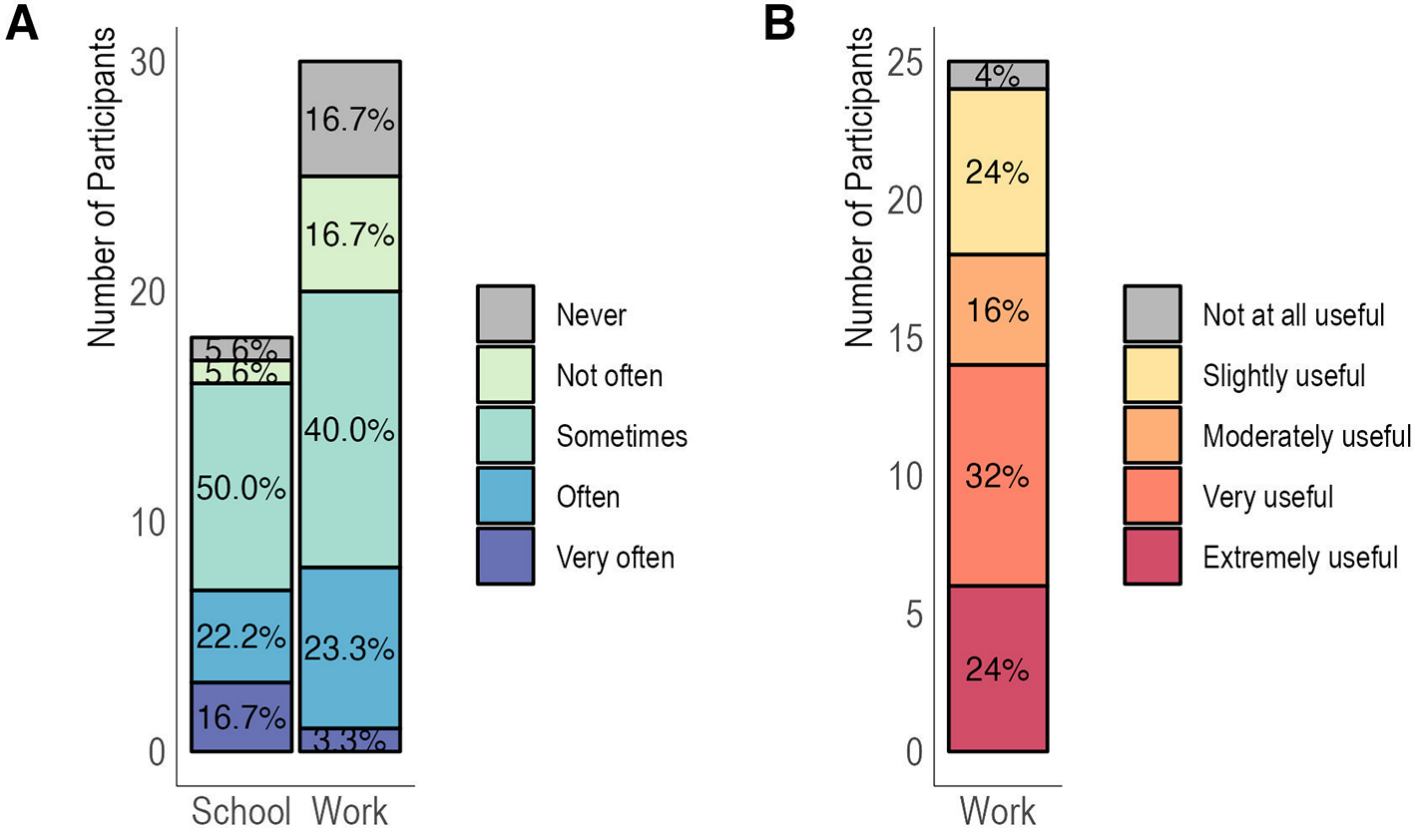
Relevance and utility of skills acquired for current job and school activities. Legend. Participants were asked about the relevance of the skills to their current academic activities, for those who reported being enrolled in a degree program, and/or to their current jobs, for those who reported being employed (Panel A). The utility of the skills was also explored for the subset of employed participants (Panel B).

Approximately 83% of the participants who indicated being employed stated that they use coding and/or biostatistics skills from the classes at work (25/30, 83.3%) ( Figure 3A). Among those applying the skills at work, 56% indicated that the skills were very or extremely useful, while 24% indicated the skills had been moderately or slightly useful for their current jobs ( Figure 3B). For the five participants who responded that they never used the skills in their jobs, nearly all (4/5) reported that tasks that require the R and statistics skills they learned were not part of their current jobs.

The relevance of the skills for academic programs was likewise high. For participants enrolled in degree programs, approximately 94% reported using coding and/or biostatistics skills from Q4D classes for school-related activities (17/18, 94.4%) ( Figure 3A).

Table 2 provides examples of how Q4D students have applied specific skills learned in the program for work or school.

**Table 2.** Examples of skills taught during the program and being applied by Q4D students.

Example	Skills [Q4D Class]
“Used my skill in R for hypothesis testing in a capacity building consultancy.”	Hypothesis testing [Biostatistics I with Intermediate R]
“When I was analyzing … Lassa Fever data [for work], I used the ggplot with the help of Google to create histogram and boxplot.”	Data visualization with ggplot2 package; Independently searching online for help [Beginner R]
“I help my uncle analyze … data he has from his pharmaceutical business. We perform all the necessary tasks and steps to make a decision. My uncle wanted to … know what medicine he can bring [to] give profit … so he can expand his investment. We used sales, product cost, shipping cost, etc as variables to investigate.”	Generating and interpreting descriptive statistics; identifying relevant independent and dependent variables from routinely collected data [Biostatistics I with Intermediate R]
“I recently used R for descriptive statistics for an article that I just submitted to a journal.”	Generating and interpreting descriptive statistics [Biostatistics I with Intermediate R]
“Coercing variables in R to different classes to perform tasks. Coercing neonatal outcome to factor from a dataset on anemia… This neonatal outcome from the dataset was in integer 1 and 2. These numbers were changed into factors such as dead and alive to analyze by outcome status.”	Determining class of an object and coercing to a new class in R; Recoding variables [Beginner R]

### Personal and professional growth

**Figure 4.**
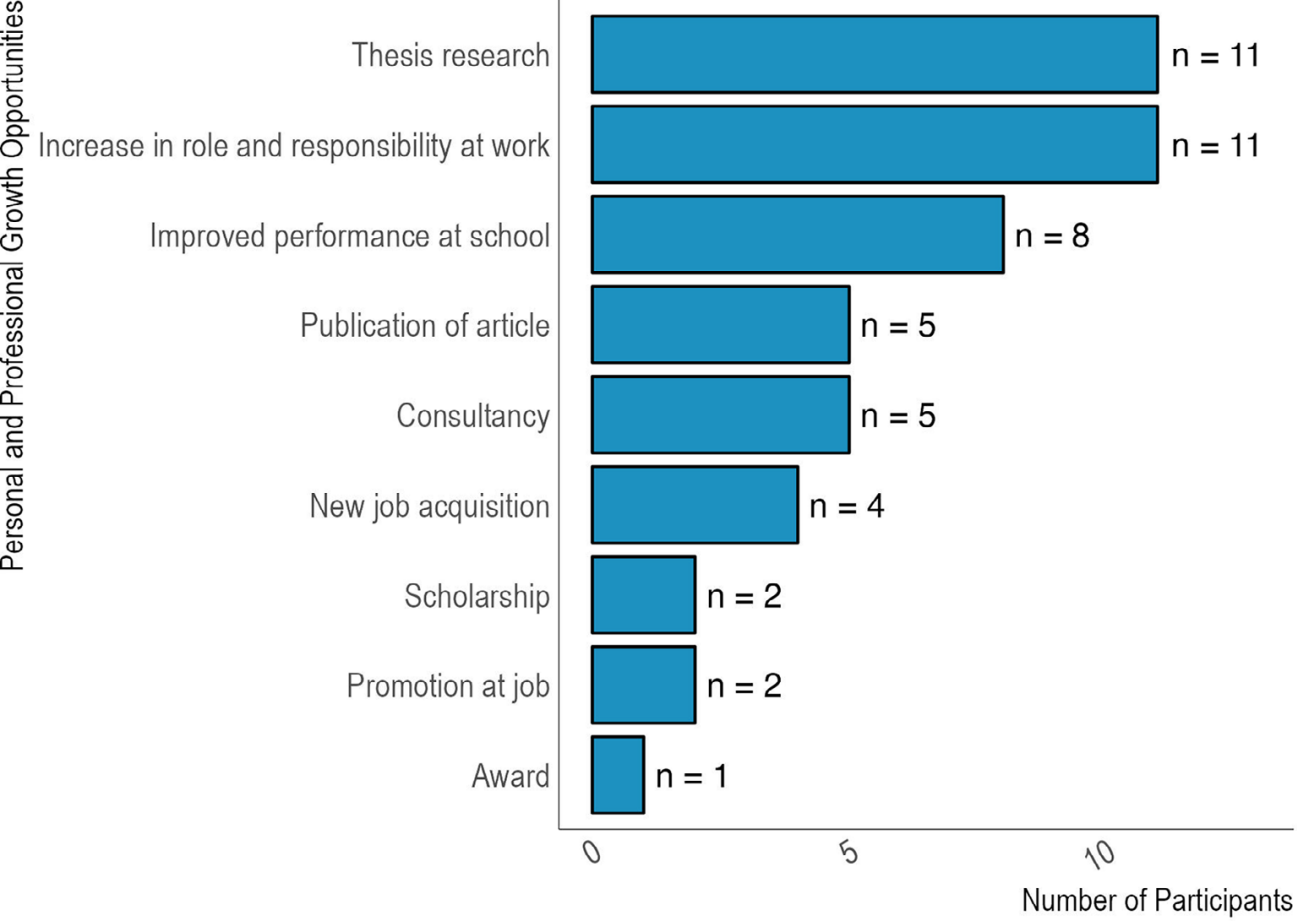
Impact of the skills on professional and academic opportunities. Legend. Survey participants indicated which opportunities they attributed to their new skills gained through the Q4D program. More than one option per participant could have been selected.

Participants reported that the skills they gained were impacting them personally through increased roles and responsibilities at work (n=11), improved performance at school (n=8), and stronger thesis projects (n=11) ( Figure 4). Several also attributed new opportunities to the skills they learned at Q4D. Five respondents indicated they had used the skills to acquire consultancies; four reported using the skills in their applications leading to new jobs; two others reported promotion at their current places of work. Other students mentioned that their Q4D course skills helped with scholarships or awards as well as publication of articles. Moreover, one-third of currently employed participants suggested that they had received an increase in income due to opportunities created with the new skills they acquired (10/30, 33.3%).

As evidence for the demand created for quantitative analysis skills and their application, all but one respondent indicated interest in pursuing a job opportunity that would utilize the coding and analysis skills taught in the program (42/43, 97.7%).

## Discussion

The findings presented here highlight how the R coding and statistics skills being taught through the Q4D program have relevance and utility to Liberians employed in health-related sectors or in the pipeline for future employment. Most of the surveyed Q4D students are confidently applying the skills they learned, and several have leveraged their new skill sets to obtain scholarships, consultancies, and promotions at work. While the present study was not a direct assessment of the skills taught (although skills evaluation is conducted as part of the courses and ahead of certification), these opportunities reflect that the capacity being built is recognized and valued by employers and/or selection committees. Moreover, our study demonstrates that students have acquired interest in quantitative research careers and that they perceive the value of quantitative research evidence for informing decision-making in the health sector. While our study is small, it offers insight into possible outcome and impact measures for assessing capacity-strengthening efforts in the quantitative sciences and lays the groundwork for larger scale evaluation as the program grows.

Increasing local buy-in for evidence-driven decision-making in health serves as a compelling case for investment in initiatives to address health workforce capacity gaps effectively. ^18^^,^^19^ Such initiatives must be two-fold—addressing the gaps to promote evidence generation and addressing the gaps to promote evidence utilization. As ongoing efforts are being undertaken to encourage research utilization in the health workforce of Liberia, ^20^ Q4D is focused on the former. Capacity-building to produce cadres of well-trained statisticians with awareness of and adherence to quality standards will not only enhance the available evidence base ^21^ but also reduce the risk of erroneous research findings that have been attributed to lack of appropriately trained statisticians. ^22^ Specifically, with increased capacity for quantitative research embedded in the health sector, analyses to assess health trends, understand factors associated with health risks, and evaluate the effectiveness of interventions will increasingly empower policymakers with rigorous evidence to make informed decisions for improved health outcomes. This result contributes to broader development objectives under the Sustainable Development Goals. ^23^ However, it is important to note that building in-depth analytical capacity in Liberia and other sub-Saharan countries is complex and warrants ongoing evaluation of context-sensitive strategies, as we have aimed to do with our tracer study.

In resource-constrained settings, quantitative research applied to routine data has the potential to extend its value and provide meaningful evidence without requiring additional cost-intensive field- or laboratory-based work. As one barrier to regular and rigorous analysis of such data, education and training programs throughout sub-Saharan Africa lack resources to develop the human capital needed for science, technology, engineering and math (STEM) fields. ^24^ In parallel, quantitative capacity-building efforts by development partners have tended to be directed towards technical assistance or short-term workshops not always sensitive to local constraints ^25^^,^^26^ or aimed at contextually relevant outcomes. ^27^

To achieve sustainable, sufficient, and well-grounded capacity in quantitative sciences, there is a clear need for improvements in fundamental STEM education across all levels. ^28^ Accomplishing this is a paradoxical situation, as building capacity where there is a lack of it requires that there are well-equipped cadres of teachers and professors across quantitative fields. ^24^ Until systemic changes can be affected, however, in-service training—such as that offered by Q4D programs—can boost confidence and capacity for coding and statistical analysis when it gauges, recognizes, and builds on the existing foundation of quantitative skills, regardless of how strong it is. Such medium-term, intensive in-service training that builds basic through advanced skills thus offers a model for demand-driven, locally based training in settings that may have a weak baseline for quantitative skills learning. Through the approach, initial progress can be made to contribute to a skilled health workforce that can address current and emerging health challenges effectively, as recommended by the World Health Organization. ^29^

While the present study suggests that the Q4D program is providing skills training in areas that are of relevance and use in students’ workplaces and/or academic programs, it was not an evaluation of the skills gained. Regular assessment is undertaken throughout the Q4D courses to ensure that students can independently demonstrate sets of pre-defined skills. One next step will be such an outcome evaluation comparing coding and analysis skills among Q4D graduates to a control group, ideally in a workplace setting. While continuing to evaluate existing efforts will be important, the present study highlights areas where the program could improve for greater impact. To promote continued opportunities for Q4D students, increased acceptance or recognition of local capacity and demand for it by health sector leadership are necessary. It has been observed that health ministries in low- and middle-income countries fail to realize their potential for knowledge transfer as learning organizations that are supportive of developing people and processes within them. ^30^ Therefore, activities by which Q4D engages health sector leadership and increases the visibility of students’ skills applications should be undertaken. To accomplish this, Q4D can learn from other local training programs, such as the Liberia Field Epidemiology Training Program, with strong ties across the health sector. Moreover, it is important that those who have completed the program and are applying the skills receive continued support to evaluate their applications for rigor (such as by adherence to statistical guidelines and standards) and to ensure ongoing exposure to additional methods. This need for in-service mentorship to extend the impact of intensive training has been noted with other programs. ^31^^,^^32^

In addition, it is recognized that the current programs offered at Q4D may be limited in scope, as they are focused on R Statistical Software and examples that largely reflect concepts from epidemiology. These design components were intentional—as R is free, open-source software, and it does not require a significant amount of space on personal computers, and as many of the initial cohorts of students were personally and professionally interested in epidemiology. However, future programs should reflect other skills and concepts to address the broader needs of the health sector. For instance, with the ongoing shift towards more transdisciplinary planetary health, ^33^ the Q4D program has experienced a higher proportion of students in or aspiring to be in the environmental health, environmental science, and/or One Health sectors. As the needs and interests of the student population evolve, project topics, datasets, and examples used in class can draw from student experiences to enhance the relevance of materials and allow for more seamless application of skills in their jobs and studies. In parallel, Q4D can align itself with other organizations that provide capacity-building in One Health or complementary areas and partner with experts from local and international universities.

## Conclusions

In conclusion, the Q4D program in Liberia offers quantitative skills training to a diverse student body, including a high proportion who are employed within the health sector. While students reported personal opportunities that arose with attainment of new skills, the program’s impact has potential to extend beyond individual growth, contributing to the country’s capacity for data-driven decision-making, research, and sustainable development. As Liberia continues to strengthen its capacity for quantitative analysis, the Q4D program could serve as a model for locally-relevant capacity building initiatives. Further evidence generation to assess skills acquisition and its use in the health sector with a larger sample size will be critical. Importantly, this focus on regular monitoring and evaluation of Q4D programmatic outcomes will ensure that ongoing programmatic decisions are evidence-based and nationally impactful. It will also set a precedent for other quantitative sciences capacity-strengthening initiatives in Liberia.

## Authors’ contributions

STB and LAS developed the initial research objectives and study protocol. MKAK, TOY provided substantive feedback on the objectives and protocol. Data collection was managed by STB and GBD and monitored by TOY and MKAK. The data analysis plan was developed by STB, TOY, MKAK, and LAS and implemented by STB and LAS. STB, GDB, and LAS developed the first draft. TOY, MKAK provided substantive feedback on the draft. All authors read and approved the final manuscript.

## Ethics and consent

The study protocol was approved by the University of Liberia IRB (ULIRB IORG-IRB Number: IRB00013730) on May 16, 2023. All study procedures were carried out according to the Declaration of Helsinki. Participants were asked to provide written consent via an online form ahead of gaining access to the questionnaire. The consent form text is included in the Extended data. All results have been presented using aggregate statistics rather than any individual participant’s response. For direct quotes on specific examples of skills applications, potentially identifying information—such as on health district where one works—was removed.

## Data Availability

Figshare, Data collection tools and dataset from tracer study of Q4D Lab, a locally developed and owned coding and biostatistics program in Liberia (doi: 10.6084/m9.figshare.26762368). ^34^ URL: https://doi.org/10.6084/m9.figshare.26762368.v2 This project contains the following underlying data:
• Q4D Reduced Dataset. A deidentified subset of the full dataset is provided. Due to small sample size and the demographic questions asked, individual participants may be identifiable by those familiar with the student population, such that some background questions on sex, degree, and title of position for those employed have been removed. Data are available under the terms of the Creative Commons Attribution 4.0 International license (CC-BY 4.0). Figshare, Data collection tools and dataset from tracer study of Q4D Lab, a locally developed and owned coding and biostatistics program in Liberia (doi: 10.6084/m9.figshare.26762368). ^34^ URL: https://doi.org/10.6084/m9.figshare.26762368.v2 This project contains the following underlying data:
• Data Collection Tools. A document with the survey tool and consent form text. Data are available under the terms of the Creative Commons Attribution 4.0 International license (CC-BY 4.0).
