# Supplementary material for: Enhancing quantitative capacity for the health sector in post-Ebola Liberia, a tracer study of a locally developed and owned coding and biostatistics program
Source: F1000Res. 2024 Sep 2;13:988. [Version 1] doi: 10.12688/f1000research.154839.1 (PMC11868749; doi:10.12688/f1000research.154839.1)
Supplement: Peer review history

# Reviewer report: Reviewer response for version 1

**DOI:** 10.5256/f1000research.169918.r365010

**Published:** 2025-02-27

**Authors:** Matthew Bonds

**Affiliations:** 1 NGO Pivot, Ranomafana, Madagascar; 2 Department of Global Health and Social Medicine, Blavatnik Institute at Harvard Medical School, Boston, USA

I enjoyed reading this. It’s a clear and coherent presentation of a coding and statistical training program in Liberia. The question driving this study is whether the training offers value to the students based on their own self-reports. According to those, there was general value provided (greater confidence, perceived application to their jobs and education). I appreciated that the survey was conducted by an independent entity to reduce biases. I nevertheless wondered whether there are internal biases inherent in such self-reports, and thus inherent limits to such a study. While I think that issue is addressed as well as it could under the circumstances, I encourage the authors to include a more substantial review of the relevant literature as background. Reading this manuscript, one is left wondering how to contextualize these results. There are many different kinds of capacity building models out there for statistical analysis and mathematical modeling across Sub-Saharan Africa. How are these programs generally assessed? Are there important differences in the structure of these trainings? What is the literature on these different models of capacity building?  Is there a basis for inferring how these trainings compare to the experiences elsewhere?

 Other than that, it’s strong work, and it’s nice to see this kind of thing published.

Is the work clearly and accurately presented and does it cite the current literature?

Yes

If applicable, is the statistical analysis and its interpretation appropriate?

Not applicable

Are all the source data underlying the results available to ensure full reproducibility?

Yes

Is the study design appropriate and is the work technically sound?

Yes

Are the conclusions drawn adequately supported by the results?

Yes

Are sufficient details of methods and analysis provided to allow replication by others?

Yes

Reviewer Expertise:

My background is in economics, quantitative epidemiology, disease ecology, infectious disease modeling, health systems strengthening

I confirm that I have read this submission and believe that I have an appropriate level of expertise to confirm that it is of an acceptable scientific standard.

# Reviewer report: Reviewer response for version 1

**DOI:** 10.5256/f1000research.169918.r321214

**Published:** 2024-10-18

**Authors:** Susan Michaels-Strasser

**Affiliations:** 1 Columbia University, New York, New York, USA

This paper presents the results of a very practical and much needed study of strengthening quantitative capacity in the health sector in under-resourced settings.  It is very well written and draws meaningful conclusions that encourage scale up of the existing Q4D efforts as well as further and more robust evaluation of the impact of the program.  

 I responded partly to one question above due to the following issues which should be easily addressed by the authors. 

 1. Figure 1.  Please make it clearer why this figure is limited to students who did the Beginner R course only.  I am not clear why you limit this figure to just them given that you run 4 courses. Is Panel B related only to the Beginner R students.  If yes, then make this clear in the figure's title.

 2. Minor issue. Page 6 paragraph 2, the statistic 16/29 (55.2%) is correct but is listed as 55.3% in the table. To avoid confusion maybe just use whole numbers in the table. 

 3. Page 7, paragraph 3. and onto page 8.  The discussion of Figure 3B needs to be edited.  You can that 24% indicated the skills had been moderately or slightly useful but the figure actually shows this total 40%. moderately useful (16%) and slightly useful (24%)

Is the work clearly and accurately presented and does it cite the current literature?

Yes

If applicable, is the statistical analysis and its interpretation appropriate?

Partly

Are all the source data underlying the results available to ensure full reproducibility?

Yes

Is the study design appropriate and is the work technically sound?

Yes

Are the conclusions drawn adequately supported by the results?

Yes

Are sufficient details of methods and analysis provided to allow replication by others?

Yes

Reviewer Expertise:

Public Health, Health Workforce Development, Competency based training and education, Epidemiology

I confirm that I have read this submission and believe that I have an appropriate level of expertise to confirm that it is of an acceptable scientific standard.
